# Enhancing implant surfaces: mechanical stability and cytocompatibility of DNase I coatings deposited by alternating current electrophoretic deposition

**DOI:** 10.3389/fbioe.2025.1738602

**Published:** 2026-01-12

**Authors:** Merve Kübra Aktan, Naiera Zayed, Aydan Yadigarli, Manuela Sonja Killian, Rob Lavigne, Wim Teughels, Annabel Braem

**Affiliations:** 1 Biomaterials and Tissue Engineering Research Group, Department of Materials Engineering (MTM), KU Leuven, Leuven, Belgium; 2 Department of Oral Health Sciences, Faculty of Medicine, KU Leuven, Leuven, Belgium; 3 Department of Microbiology and Immunology, Faculty of Pharmacy, Menoufia University, Shebin El-Kom, Egypt; 4 Chemistry and Structure of Novel Materials, University of Siegen, Siegen, Germany; 5 Laboratory of Gene Technology, Department of Biosystems, KU Leuven, Leuven, Belgium

**Keywords:** coatings, cytocompatibility, deoxyribonuclease (DNase), electrophoretic deposition (EPD), mechanical stability

## Abstract

Deoxyribonuclease I (DNase I) is an enzyme that hydrolyzes the phosphodiester bonds in the DNA backbone, enabling efficient DNA degradation. This activity is particularly relevant for degrading extracellular DNA (eDNA), a key structural component of the biofilm extracellular matrix that enhances bacterial attachment to implant surfaces and promotes cell-to-cell adhesion. By disrupting this matrix, DNase I offers significant potential to indirectly inhibit biofilm formation and reduce the risk of implant-associated infections (IAIs). In previous work, we developed a rapid electric field-assisted technique for producing anti-infective DNase I coatings on titanium (Ti) implant surfaces. To further evaluate clinical applicability, this study investigates the mechanical stability and *in vitro* cell compatibility of DNase I coatings applied to polydopamine (PDA)-functionalized Ti. Coatings were mechanically stressed using ultrasonication, followed by characterization of surface wettability and chemical composition. Compared to traditional dip-coating, AC-EPD-generated DNase I coatings exhibited greater stability, maintaining consistent wettability after 2 h of ultrasonication. Surface chemistry was examined using time-of-flight secondary ion mass spectrometry (ToF-SIMS), which detected amino acid fragments on both coating types. Notably, AC-EPD coatings contained a higher disulfide bond content, suggesting enhanced structural integrity. Furthermore, given the relevance to dental implant applications, human oral keratinocytes (HOKs) were used to assess cytotoxicity, cell adhesion, and spreading. The results indicated no cytotoxic effects, while promoting improved cell adhesion at both 24-h and 48-h incubation periods. Overall, these findings demonstrate that AC-EPD DNase I coatings are mechanically robust and biocompatible, making them a promising strategy for preventing implant-associated infections (IAIs) in dental implant applications.

## Graphical Abstract

**Figure:**
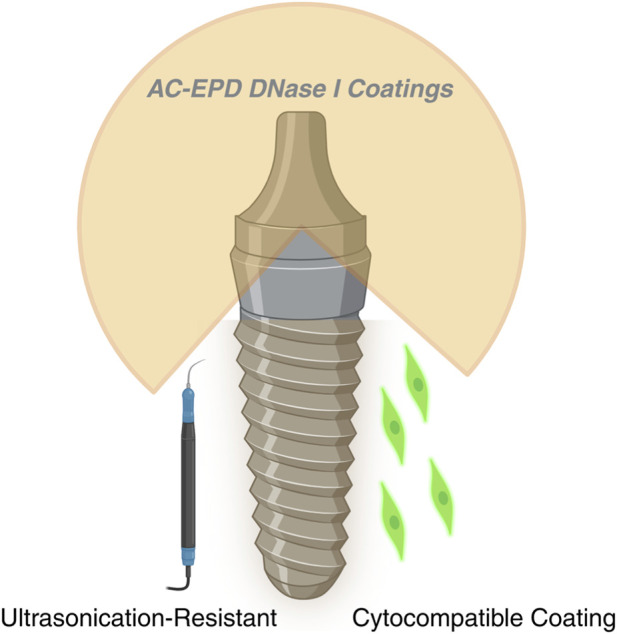

## 1 Introduction

Implant-associated infections (IAIs), being one of the major causes of orthopedic and dental implant failure, pose a huge problem to patient safety and healthcare economics. IAIs are commonly resulting from microbial biofilms, complex ecosystems of microorganisms embedded in a self-produced extracellular polymeric substance (EPS) consisting of polysaccharides, proteins and extracellular deoxyribonucleic acid (eDNA), which form on the implant surface (Tan et al., 2021; Benčina et al., 2021). In this catastrophic complex, which can threaten the entire implant surface, microorganisms remain protected from exposure to internal host defense mechanisms or external stresses, such as antibiotic treatment. However, anti-infective implant surfaces by means of antimicrobial compounds/molecules, such as antibiotics (Davidson et al., 2015; Nichol et al., 2021; Brown et al., 2012), antimicrobial peptides (Shi et al., 2015; Braem et al., 2017; Cado et al., 2013), enzymes (Lauková et al., 2020; Ye et al., 2017; Aktan et al., 2022a; Swartjes et al., 2013), polysaccharides (Tan et al., 2013; Afroz et al., 2021; Ke et al., 2021; Kobata et al., 2020) can prevent or at least decrease biofilm formation and thereby reduce the incidence of implant failure. Even though antibiotics are well-known as antimicrobial agents, there is a rising concern regarding their use, because microbes can acquire resistance to the drugs, which eventually poses an even bigger risk for the treatment of infections (Høiby et al., 2010; Roilides et al., 2015). Alternatively, antimicrobial coatings based on biomolecules, particularly those that allow targeting the biofilm matrix, have been gaining interest due to their non-toxic nature and possibility to avoid antimicrobial resistance phenomena (Pinto et al., 2020; Ghalayani Esfahani et al., 2019). Deoxyribonuclease I (DNase I) is an enzyme which is capable of hydrolyzing the phosphodiester bonds of DNA molecules, such as the eDNA found in biofilm matrices (Lauková et al., 2020). Since eDNA plays a vital role in the adsorption of microorganisms to abiotic surfaces or as intercellular connector holding microbial colonies together, but also in antibiotic resistance development by transferring knowledge between sessile colonies, targeting eDNA is a highly interesting approach to sensitize or eradicate biofilms (Sutherland, 2001; Okshevsky et al., 2017). As such, we have previously investigated the fast processing of DNase I coatings on titanium (Ti) by means of alternating current electrophoretic deposition (AC-EPD) (Aktan et al., 2022a). It was shown that, after polydopamine (PDA) functionalization of the Ti electrodes, AC-EPD allowed concentrating DNase I more on the Ti surface than a simple dip-coating procedure, which resulted in a superior enzymatic activity and a significant reduction of biofilm formation of both *Staphylococcus epidermidis* and *Pseudomonas aeruginosa*. This allowed us to establish a proof-of-concept for AC-EPD DNase I coatings for anti-infective applications. However, to further assess its potential for implant applications, both mechanical stability during storage and implantation, and safety in contact with host tissues, need to be evaluated.

In order to guarantee a long-term bioactive effect of biomolecules, coatings should remain at the implant surface with preservation of their activity during manipulations inherent to the intended application (e.g., sterilization, storage and implantation procedures). Durable attachment of biomolecule coatings is often established by immobilization, i.e., covalent attachment, of the molecule on the implant surface (Holmberg et al., 2013; Koidou et al., 2018). To this end, PDA, the polymerized form of dopamine (DA), was used to pretreat the Ti electrodes prior to AC-EPD (Aktan et al., 2022a; Aktan et al., 2022b). This versatile, non-expensive surface functionalization strategy allows introducing a large number of functional groups on the surface for covalent grafting, such as catechols, quinones and primary amines (Alfieri et al., 2018). Several studies have highlighted that an intermediate PDA layer significantly improves the immobilization capacity and furthermore increases the biomolecule stability on the surface (Trzcińska et al., 2020; Dinh et al., 2018; Zhang et al., 2016). In our previous study, it could be seen that DNase I coatings still retained significant enzymatic activity upon storage at 4 °C for 1 week. Although the shelf life of AC-EPD DNase I coatings showed promising results, gently executed release testing indicated a rapid burst release of a large amount of the DNase I (Aktan et al., 2022a). Even though a remaining surface activity could be observed, coating stability testing using more harsh mechanical challenges is required.

Cytocompatibility of the AC-EPD DNase I coatings is another important aspect that needs to be addressed. The coatings should not induce any harmful effects on the healthy host tissues with which they will be in contact. DNase I is considered safe in human applications, as the recombinant human DNase I was commercialized to treat the lung disease of cystic fibrosis (Lauková et al., 2020; Yang and Montgomery, 2018). In previous reports, DNase I coatings obtained by dip-coating did not induce any adverse effects on the cell structure of human osteosarcoma (U2OS) and MC3T3-E1 osteoblast-like cells (Ye et al., 2017; Swartjes et al., 2013). Moreover, DNase I coatings appeared to enhance cell adhesion and growth (Ye et al., 2017). This suggests a good cytocompatibility of dip DNase I coatings, yet, the cytocompatibility of DNase I coatings specifically using AC-EPD has not yet been evaluated.

In this study, we evaluate the mechanical stability and cytocompatibility of AC-EPD DNase I coatings on PDA-functionalized Ti surfaces. With respect to mechanical stability, coatings will first be challenged by ultrasonication followed by characterization of possible changes in wettability via contact angle measurement or surface chemistry via time-of-flight secondary ion mass spectroscopy (ToF-SIMS). Next, *in vitro* cell culture tests using human oral keratinocytes (HOK) will be performed on control and DNase-treated surfaces and cell viability, cell adhesion/morphology and proliferation of HOK cells will be evaluated using XTT assay, confocal laser scanning microscopy (CLSM) using immunofluorescence staining and scanning electron microscopy (SEM).

## 2 Materials and methods

### 2.1 Preparation of DNase I coatings

#### 2.1.1 Substrate preparation

Commercially pure (cp) Ti discs (grade 2, Ø = 5 mm, thickness = 2 mm; Biotech Dental) were prepared by grinding with an automatic polishing machine (Struers) equipped with resin-bonded diamond grinding discs (MD-Mezzo 220 and MD Largo; Struers) and by polishing with an Oxide Polishing Suspension (OPS; colloidal silica, Microdiamant) and hydrogen peroxide (H_2_O_2_; 35 wt%, Chem-Lab) mixture (70:30 by vol%) on a synthetic polishing cloth (Galaxy Polishing Cloth Omega; Advanced Metallography). All samples were cleaned with acetone (>99%, Chem-Lab) for 20 min in an ultrasonic bath (model 2510, Branson) followed by extensive rinsing with ultra-pure water (milliQ, 18.2 μs/cm, Merck Millipore) and technical ethanol (Disolol, Chem-Lab). These pristine polished Ti substrates (pol-Ti) were further functionalized with a PDA layer by immersing them for 24 h in a 0.5 mg/mL dopamine (DA, 99%; Alfa Aesar) solution in Tris buffer (10 mM, pH 8.5; Acros Organics) in an incubator (IKA KS 4000 i control) at 22 °C in the dark (Aktan et al., 2022b). Afterwards, the samples were ultrasonically cleaned with milliQ for 5 min. The resulting PDA-functionalized Ti substrates (pDA-Ti) were left to dry under a fume hood and finally stored at 4 °C until further use.

#### 2.1.2 Alternating current electrophoretic deposition of DNase I

**FIGURE 1:**
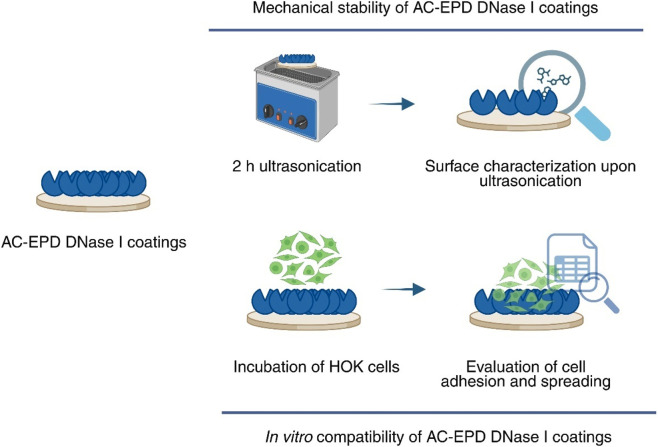
Schematic representation of the evaluation protocol for AC-EPD DNase I coatings. Discs were ultrasonicated for 2 h and the mechanical stability of the AC-EPD DNase I coatings was assessed by wettability measurement and time-of-flight secondary ion spectrometry (ToF-SIMS) analysis. To prove the cytocompatibility of the coatings, cytotoxicity as well as the proper functioning (adhesion and spreading) of human oral keratinocytes (HOK) on AC-EPD DNase I coatings was addressed *in vitro*.

Aqueous suspensions of 0.0125 mg/mL deoxyribonuclease I from bovine pancreas Type IV (DNase I, 2814 U mg-1, D5025; Sigma-Aldrich) were prepared fresh in milliQ by gentle pipetting before every experiment. AC-EPD was carried out in the controlled-current mode under ambient conditions, as described previously (Aktan et al., 2022a). A schematic representation of the deposition cell is given in Figure 1. Custom-made Ti clips were placed around the pDA-Ti discs to ensure electrical contact along the side of the discs and two identical electrode systems were inserted in polystyrene cuvettes (Macrocuvette, VWR). The electrodes were placed vertically at a fixed interelectrode distance of 0.8 cm using a polytetrafluorethylene spacer. As in our earlier work, an asymmetrical triangular AC signal with an asymmetry of 3 and a frequency of 100 Hz was generated using a high-resolution digital-to-analog signal output module (NI9269, National Instruments) and amplified with a bipolar amplifier (PZD 7000 M S-1, Trek Inc) (Abedi and Pourmohammadi, 2021). A peak-to-peak current of 6 mA/cm^2^ was applied for 10 min. Current and voltage were monitored by an analog-to-digital acquisition module (NI9223, National Instruments) linked to the monitor channels of the operational amplifier. Afterwards, the substrates were removed from the cuvette and rinsed by gently dipping in milliQ followed by drying at room temperature. AC-EPD coating samples are denominated as DNase-EPD, this refers to the electrodes used as anode during the high-amplitude peak unless otherwise specified. For comparison, a classic dipping experiment was conducted for 10 min using the same DNase I suspension as well as the same deposition cell. Samples, denominated as DNase-dip, were rinsed and dried in the same way as the DNase-EPD samples.

### 2.2 Mechanical stability testing

#### 2.2.1 Mechanical challenge

Discs were placed individually in a 50 mL plastic tube containing 10 mL of milliQ and sonicated for 2 h. Following sonication, samples were rinsed with milliQ and allowed to dry in atmospheric conditions.

#### 2.2.2 Wettability

The surface wettability before and after sonication was analyzed using an Optical Contact Angle and Contour Analysis system (OCA 15EC, Dataphysics) applying the sessile drop method for static contact angle measurements using milliQ. Contact angles were captured employing a video system and determined using a software-operated fitting procedure (SCA 20 software, Dataphysics).

#### 2.2.3 Time-of-flight secondary ion mass spectrometry (ToF-SIMS)

To evaluate the surface chemistry of the Ti substrates after several surface treatments, positive and negative static SIMS measurements were recorded on a ToF-SIMS spectrometer (ToF.SIMS 4, ION-TOF). Spectra were recorded in the high mass resolution mode using a pulsed 25 keV Bi^3+^ liquid-metal ion beam in static conditions (primary ion dose density = 5 × 10^11^ ions cm^-2^). Poisson corrected spectra were calibrated to CH_3_^+^, C_2_H_3_^+^, C_3_H_5_^+^, C_4_H_9_^+^ and C_7_H_7_^+^ (C^−^, CH^−^, CH_2_^−^ and C_2_^−^ for negative polarity, respectively). None of the spectra showed artefacts from ion beam instabilities (e.g., peak broadening, low total spectral intensity). The “Spectragui” software from the NESAC/BIO toolbox was applied for principal component analysis (PCA) (National Institute for Biomedical Imaging and Bioengineering NIBIB, 2022). The peak set used for PCA processing of the positive spectra is described in the Supplementary Material. A peak list containing amino acid (AA), PDA and substrate fragments was compiled according to literature (Killian et al., 2018). The PDA and substrate fragments were identified by comparing PDA to DNase or Ti samples via PCA. The total intensity of the PCA peak set was used for spectra normalization and square root mean-centering was used to ensure the differences in samples are caused by variations around the means and not the variance of the means (Massart et al., 1998). Salt and substrate signals have been removed for the PCA analysis comparing only the samples that were in contact with DNase.

### 2.3 *In vitro* cell compatibility study

#### 2.3.1 Cell cultures

Immortalized human oral keratinocytes (HOK-18A) were grown in tissue culture flasks using keratinocyte serum-free medium (KSFM, Gibco) supplemented with bovine pituitary extract (25 μg/mL) and recombinant epidermal growth factor (5 ng/mL). The cells were maintained in a humidified incubator with 5% CO_2_ and 95% air at 37 °C. For further experiments, cells were collected from the flasks by trypsinization.

#### 2.3.2 Cytotoxicity assay

Silicon rings (Peleman bvba) were placed at the bottom of 48- or 24-well tissue-culture plates (Greiner Bio-One) before cells were seeded at a concentration of 40,000 or 80,000 cells per well in 300 or 500 µL of culture medium, respectively, and incubated until confluence as observed under a microscope. After a state of confluence was reached, the culture medium was removed from each well and replaced by fresh medium. Next, the cells were exposed to the tested coated Ti discs. Wells without Ti discs served as controls, with negative control wells only containing KFSM, whereas in positive control wells 1% Triton X-100 was added to the KSFM. 1 µL/100 µL of 1 mM Ca^2+^ and 10 mM Mg^2+^ ions solutions were also supplemented to the medium. After 20 h incubation, the discs were removed from the well and an XTT assay (Sigma Chemical Co.) was performed to assess the cell viability. Briefly, 2,3-bis (2-methoxy-4-nitro-5-sulfophenyl)-2H-tetrazolium-5-carboxanilide sodium salt (XTT) was dissolved in RPMI 1640 medium (Life Technologies) at a concentration of 1 mg/mL, while phenazine methyl sulphate (PMS) powder (AppliChem) was dissolved in phosphate buffered saline (PBS) at a concentration of 0.383 mg/ml. A mixture of 150 μL XTT + 3 μL PMS or 250 μL XTT + 5 μL PMS was added to each well of the 48- or the 24-well plates, respectively, after removing a similar volume of the old medium. Cells were then incubated for 4 h at 37  C and afterwards, the concentration of formazan, which is released by the cells and serves as indication of cell viability, was determined by optical density (OD) measurements at 450 nm after subtracting the background signals at 630 nm using a micro-plate reader (Thermo Scientific). Cell viabilities are expressed as the percentages with respect to the negative control which was prepared without any Ti substrates.

#### 2.3.3 Cell adhesion and proliferation study

To investigate the effect of different Ti surface conditions on host cell adhesion, HOKs were grown in the disc surface and visualized by confocal laser scanning microscopy (CLSM) following live/dead staining as well as scanning electron microscopy (SEM) in order to assess spreading of the cells and the presence of cellular junctions. To this end, HOKs were collected from the tissue culture flasks and seeded into the well plates containing the various Ti discs. To allow for cell attachment and growth on the disc surface, discs were incubated for 24 h or 48 h. Afterwards, discs were collected and rinsed by gentle dipping in PBS, then the cells were fixed by immersing the samples in a 4% paraformaldehyde aqueous medium for 30 min at room temperature. Next, samples were washed with PBS after which cells were permeabilized by immersion in an aqueous 0.5% TritonX-100 solution for 10 min at room temperature followed by washing with PBS and incubation in an aqueous 2% bovine serum albumin (BSA) solution for 30 min at room temperature to block the unspecific protein binding sites. Afterwards, actin (FITC Phalloidin, Hello Bio Limited) and nuclear (Hoechst 33342, Hello Bio Limited) staining were applied at room temperature for 20 min and 15 min, respectively, with a PBS washing step in-between. Finally, the samples were washed with PBS, dipped once in milliQ water and placed with the coatings and cells facing down on a microscopic 1.5 glass coverslip followed by embedding with a Mowiol mounting solution (Mowiol® 4-88, Sigma-Aldrich) and overnight drying. CLSM visualization was performed on an inverted Leica true confocal scanner SP8 X system (Wetzlar) equipped with a HC PL APO ×10 dry objective (NA 0.4) using the green and red fluorescence channels. For each replicate sample, images were acquired for at least three different positions with an image size of 1024 × 1024 pixel^2^, corresponding to a pixel size of 1.14 × 1.14 μm^2^. Besides a qualitative evaluation based on the CLSM images, HOK cell attachment and spreading was also quantified by counting of the Hoechst-positive nuclei as well as the total phalloidin-positive actin area, respectively. Analysis was performed using an image processing software (Fiji, ImageJ) applying an automatic threshold protocol (Huang’s method) for images at equal gamma, brightness and time exposure settings, and this for a total of 3 images per replicate. Reported values are an average of nine measurements distributed over three replicate samples. Alternatively, to CLSM imaging, SEM visualization was applied for assessing the cell adhesion at higher magnifications. Therefore, after the cells were grown on the disc surfaces for 24 h or 48 h, the discs were collected and washed with PBS. Next, adherent cells were fixed using a 2.5% glutaraldehyde solution in cacodylate buffer (0.1 M pH 7.4) for 30 min. Afterwards, the discs were removed from the glutaraldehyde solution and rinsed with PBS. Dehydration was performed through a series of ethanol exposures (30, 50, 70, 90%) for 20 min each. Then, the discs were soaked in 100% ethanol three times for 20 min each. Finally, the discs were allowed to dry in atmospheric conditions followed coating with 5 nm Pt layer using a sputtering device (Q150/S, Quorum Technologies). The cells were then visualized by SEM (Nova NanoSEM 450, FEI) operated at high-vacuum settings using a backscattered electron detector. A low accelerating voltage (5 keV) was used to minimize beam damage on the cells, and the working distance and spot size were set to 5 mm and 3, respectively.

## 3 Results and discussion

### 3.1 Mechanical stability of DNase I coatings

DNase I coatings prepared by AC-EPD or dip-coating were mechanically challenged by 2 h ultrasonication, after which surface wettability (contact angle) and surface chemistry (ToF-SIMS) were analyzed.

**FIGURE 2:**
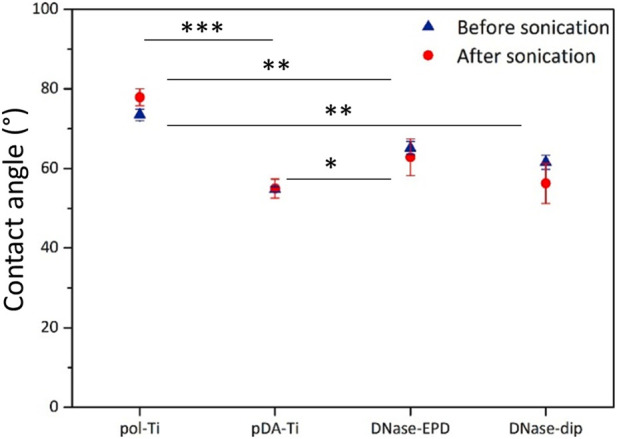
Surface wettability before and after ultrasonication. Water contact angles before and after 2 h of ultrasonication for pristine polished titanium (pol-Ti), functionalized with an intermediate PDA layer (pDA-Ti) and coated with DNase I by means of AC-EPD (DNase- EPD) or classic dipping (DNase-dip). Data are presented as average ± standard deviation with n = 4 samples per condition. Statistical significance is indicated using the following notation: p < 0.05 (*), p < 0.01 (**), and p < 0.001 (***).

For comparison, DNase I coatings obtained by immersion were included, as well as control surfaces of pol-Ti and pDA-Ti. Upon sonication, temperature was measured as 36.8 °C, meaning that sonication elevated the temperature of sample medium. To reveal possible effects of the ultrasonication treatment, the surfaces were first characterized for wettability by water contact angle (CA) measurements (Figure 2). The observed contact angles remained largely the same for all samples before and after ultrasonication, indicating all moderately hydrophilic surfaces. Statistical comparison of the upon sonication measurements confirmed that pol-Ti exhibited significantly higher contact angles than PDA-Ti, DNase-EPD, and DNase-dip (p < 0.01), whereas no significant differences were observed between pDA-Ti and DNase-dip or between DNase-EPD and DNase-dip. DNase-EPD showed a small but statistically significant difference compared to PDA-Ti (p = 0.038).

**FIGURE 3:**
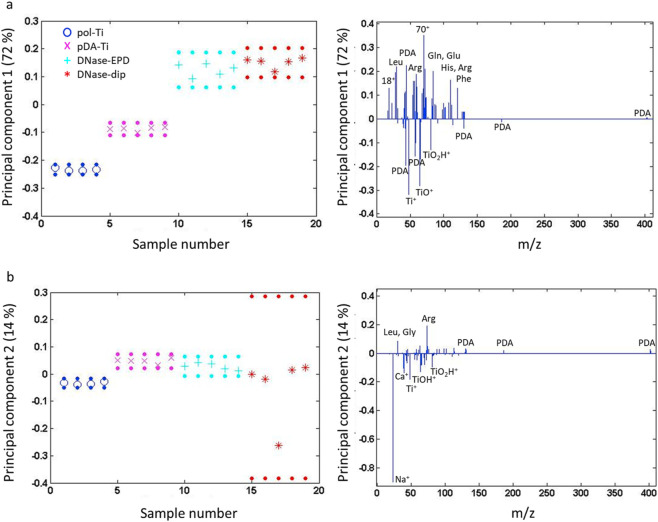
PCA results of ToF-SIMS spectra for control and DNase-coated samples after 2 h of sonication. **(a)** Scores plot (left) with corresponding loadings plots (right; all signals above a threshold of 0.1 are labeled, signals corresponding to multiple fragments are labeled with their mass) of **(a)** PC1 and **(b)** PC2 for pristine titanium (pol-Ti), PDA-functionalized titanium (pDA-Ti), PDA-functionalized titanium substrates after treatment with DNase I by means of AC-EPD (DNase-EPD) or by classic dipping (DNase-dip). PC1 separates pol-Ti and pDA-Ti s from the DNase I treated samples. i.e., DNase-EPD and DNase-dip samples. PC2 separates pol-Ti from pDA-Ti and DNase I treated samples (DNase-EPD, DNase-dip (except one point)).

Furthermore, ToF-SIMS measurements in combination with PCA were conducted to observe possible changes in surface chemistry upon sonication. A peak list containing characteristic fragments of proteins, PDA and the Ti substrate were composed (see Supplementary Table S1) and the positive polarity ToF-SIMS spectra of the various samples were fitted with it and subjected to PCA. First, the different surface conditions after sonication were compared. Figure 3 shows principal components (PC) 1 and 2, capturing a combined spectral variance of 86%. A remarkable change in the surface chemistry could be distinguished after the various steps. In PC1, pol-Ti and pDA-Ti were separated from samples which were in contact with DNase I. The characteristic fragments of the pol-Ti substrate and PDA loaded negatively in PC1, while all protein related fragments and a few PDA related components loaded positively in PC1 (Figure 3a). DNase-coated samples, i.e., DNase-EPD and DNase-dip, contain amino acid fragments such as leucine (Leu), arginine (Arg), glutamine (Gln), glutamic acid (Glu), histidine (His) and phenylalanine (Phe). Moreover, PC2 clearly separated pol-Ti from the other samples except one point for DNase-dip, which can be attributed to the presence of salt ions, such as Na^+^ and Ca^2+^ (Figure 3b). Overall, the results confirm that both DNase-treated samples (DNase-EPD and DNase-dip) still contain amino acid fragments, indicating that DNase I is still present at the Ti surface after ultrasonication. This is in accordance with the result of the release test performed in our previous study and is even more remarkable as the mechanical challenge was more severe in the current investigation. Since the effect is observed for both DNase-EPD and DNase-dip, it can be explained by the effective covalent bonding of a monolayer of DNase molecules on the intermediate PDA layer. Whereas both coatings might consist of multiple layers of DNase I weakly adhered by physical adsorption (but with higher thickness in case of DNase-EPD), the final covalently bound layer remains stable at the surface even upon ultrasonication. It is unclear from these results, however, whether the enzymatic activity and hence the antimicrobial effect of the coatings is preserved. Therefore, it is suggested to compare the enzymatic activity of both DNase-treated surfaces after 2 h sonication via the qDNase assay (Van der Gucht et al., 2021).

**FIGURE 4:**
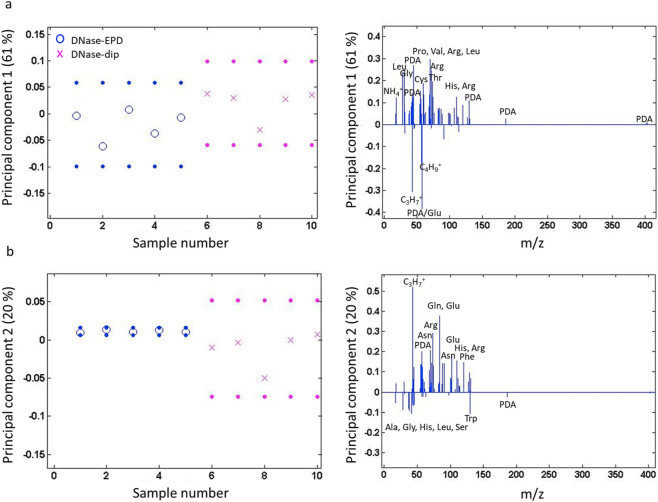
PCA results of ToF-SIMS spectra for DNase-coated samples after 2 h of sonication. Scores plot (left) with corresponding loadings plots (right; all signals above a threshold of 0.1 are labeled, signals corresponding to multiple fragments are labeled with their mass) for **(a)** PC1 and **(b)** PC2 for PDA-functionalized titanium substrates after treatment with DNase I by means of AC-EPD (DNase-EPD) or by classic dipping (DNase-dip) followed by 2 h sonication.

For a more detailed comparison of DNase-coated surfaces, a PCA was conducted only taking into account DNase-EPD and DNase-dip; salt and substrate signals were removed from the peak list used (Figure 4). In PC1, main variance resulted by PDA-related fragments and some aromatic fragments which could possibly be fragments of a PDA layer (Figure 4a). Compared to previous findings in DNase I coating procedure (Aktan et al., 2022a), this observation supports that DNase I coatings are deformed to be a thinner or fragmentary protein coating due to the sonication. In PC2 (Figure 4b), DNase-EPD showed a less variance, and loaded positively, while DNase-dip exhibited a heterogeneity with a negative loading. These differences between DNase-treated samples could be that dip coating results in a monolayer formation which can be disturbed during the sonication and lead to a patchier protein layer.

**FIGURE 5:**
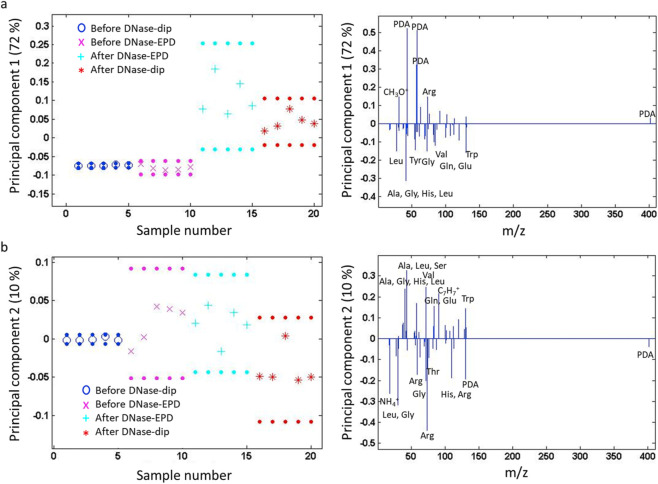
PCA results of ToF-SIMS spectra for DNase-coated samples before and after 2 h of sonication. Scores plot (left) with corresponding loadings plots (right; all signals above a threshold of 0.1 are labeled, signals corresponding to multiple fragments are labeled with their mass) for **(a)** PC1 and **(b)** PC2 for PDA-functionalized titanium substrates after treatment with DNase I by means of AC-EPD (DNase-EPD) or by classic dipping (DNase-dip) and DNase-EPD and DNase-dip samples followed by 2 h sonication.

Lastly, another PCA was performed to compare DNase-treated samples before and after 2 h sonication (Figure 5). The PC1 showed clear amino acid fragments on fresh DNase-treated samples before sonication (Figure 5a), however, more PDA fragments were observed on DNase-treated samples upon sonication. The signals related to the amino acid fragments loaded negatively in PC1, while PC2 indicated differences in amino acid fragments as they loaded in both polarities (Figure 5b). Regardless of sonication, all samples that encountered DNase I contain amino acid fragments, however, with different content. For instance, alkaline amino acids such as arginine (Arg) were prominent on DNase I-coated samples upon sonication, while hydrophobic amino acids such as leucine (Leu), alanine (Al), and valine (Val) were dominant on fresh DNase I-coated samples. Arginine has a crucial role in forming salt bridges via interaction with acidic amino acids (i.e., Glu) to determine the protein structures, and protein-protein interactions (Xie et al., 2015). The presence of exposed alkaline amino acids on the surface can be the reason for the slight reduction of hydrophobicity after 2 h sonication (Figure 2).

The PCA results presented above are based on the positive polarity spectra which yields information about the characteristic amino acid fragments. Yet, negative polarity spectra can provide information about the tertiary structure of proteins (Killian et al., 2011). The tertiary structure of proteins relies on disulfide bonds which arise from the interaction of two cysteine residues, along with hydrogen bonds and van der Waals interactions. The intensity of the disulfide S_2_^−^ (m/z = 63.94) signal, relative to the CNO^−^ (m/z = 42.00) signal originating from the peptide backbone of DNase I, is a measure for the stability of the tertiary structure and thus absence of denaturation. Compared to previous work (Aktan et al., 2022a), the disulfide bond signal (S_2_^−^ values relative to the peptide bond CNO^−^) for DNase-dip reduced by 17.1% after sonication (0.0070 ± 0.0003 vs. 0.0058 ± 0.0002), pointing at some degree of denaturation. However, for DNase-EPD, the disulfide bond signal was significantly increased by 377.7% (0.0072 ± 0.0003 vs. 0.0272 ± 0.0096) with sonication. It has been reported before for goose liver proteins that ultrasonication increased the disulfide bond content (Zhao et al., 2019), owing to the oxidation of unstable sulfhydryl (SH) groups of the protein by the reactive species originating from the dissociation of water during sonication (Zhang et al., 2018; Abedi and Pourmohammadi, 2021). However, since this effect was not observed for DNase-dip, an alternative explanation could be the re-aggregation of physically adsorbed DNase I molecules leading to the formation of extra disulfide bridges (Zhao et al., 2019).

Biomolecule coatings are an interesting strategy to confer bioactive properties to the otherwise inert Ti implant surface. Covalent immobilization, i.e., grafting through irreversible chemical bonds, is commonly considered as the best method for a stable attachment of the biomolecules at the implant surface (Holmberg et al., 2013; Tejero et al., 2014). As such, antimicrobial agents, such as antimicrobial peptides or enzymes, were covalently grafted to provide prolonged activity (Ye et al., 2017; Kucharíková et al., 2016; Swartjes et al., 2013). However, these soft biological layers can be easily damaged by physical abrasion during or after implantation or by shear stresses from fluid flow, leaving the exposed surface susceptible to bacterial attachment and subsequent biofilm formation (Abdallah et al., 2017).

For a long-term antimicrobial effect and thus protection against infections, a durable homogeneous surface coverage of biomolecules is required. In our previous work, we have shown that AC-EPD allows producing dense and homogeneously distributed DNase I coatings on PDA-functionalized Ti. Moreover, during release testing for 24 h, it could be observed that some enzymatic activity was still present at the Ti surface, suggesting that at least part of the coating remained more strongly anchored at the surface (Aktan et al., 2022a). This might be explained by the tethering of the molecules to the intermediate PDA layer, which has been shown to improve the stable bonding of biomolecules before (Zhang et al., 2016; Zhu et al., 2019). Here, we aim to investigate the mechanical stability of the AC-EPD DNase I coatings in more detail.

There is a variety of methodologies used in literature to evaluate the stability of biomolecule coatings going from simple immersion in buffered or in aqueous media at 4 °C (Aktan et al., 2022a), or at room temperature (Zhu et al., 2019), or at 37 °C (Trzcińska et al., 2020; Zhang et al., 2016) to mechanically challenging the coatings using ultrasonication (Holmberg et al., 2013; Koidou et al., 2018), scratch testing (Lewis and Mantovani, 2009) and even inserting in artificial bone material (e.g., Sawbone®) (Shiel et al., 2020). All the listed methods apply a harsh environment to challenge soft coatings, however, do not directly mimic the real situation. Yet, the mechanical challenging of peptide coatings using ultrasonication can suggest that it can mimic the environment during the surgical placement or the *in vivo* fluid flow forces (Holmberg et al., 2013).

### 3.2 *In vitro* cell compatibility of DNase I coatings

To assess how DNase I coatings may interact with host tissue, their effect on HOK cell behavior was evaluated, with pristine pol-Ti and pDA-Ti included as controls to isolate the impact of the coating.

**FIGURE 6:**
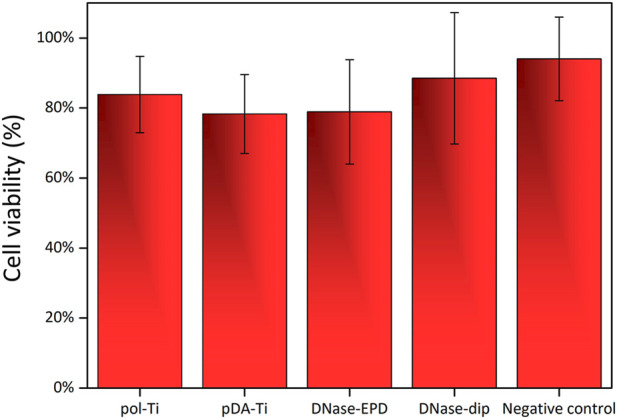
Cytotoxicity testing for human oral keratinocytes in contact with DNase-coated samples. Cell viability assessed by XTT for human oral keratinocytes (HOKs) after 24 h of direct contact with pristine polished titanium (pol-Ti), functionalized with an intermediate PDA layer (pDA-Ti) and coated with DNase I by means of AC-EPD (DNase- EPD) or classic dipping (DNase-dip). Well-plate without any Ti substrates serves as negative control. Data are presented as average ± standard deviation as percentage relative the negative control. For each condition, the number of samples was n = 5, and statistical analysis showed no significant differences in cell viability among the tested Ti-based surfaces.

Firstly, cytotoxicity was addressed by measuring cell viability using an XTT assay (Figure 6). After 24 h of incubation, cell viability appeared similar for all samples, no significant differences between samples were observed. Contrastingly, a previous study by Ye et al. revealed a slight improvement of MC3T3-E1 cell viability on DNase I dip-coated on Ti in comparison to both polished Ti or PDA-functionalized Ti (Ye et al., 2017). Although, it is not straight forward to compare previous studies with our current findings due to several experimental differences (i.e., cell and assay type, coating procedure difference).

**FIGURE 7:**
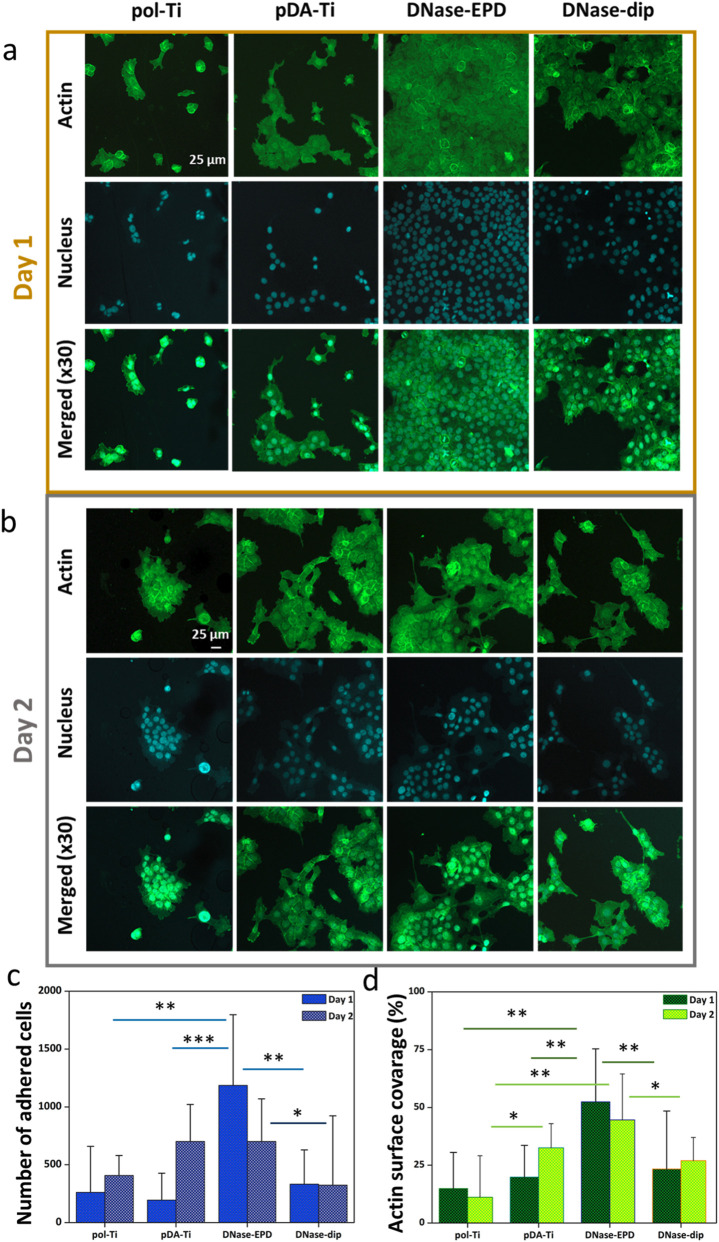
Representative CLSM images of human oral keratinocytes on DNase-coated samples after 24 h and 48 h incubation. Actin cytoskeleton (left), cell nuclei (middle), and merged (right) images of human oral keratinocytes (HOKs) after **(a)** 24 h or **(b)** 48 h of incubation on pristine polished titanium (pol-Ti), functionalized with an intermediate PDA layer (pDA-Ti) and coated with DNase I by means of AC-EPD (DNase- EPD) or classic dipping (DNase-dip). Cell number and surface coverage of human oral keratinocytes on DNase-coated samples after 24 h and 48 h incubation. **(c)** Total number of cell nuclei and **(d)** actin surface coverage, as calculated from CLSM images, for human oral keratinocytes after 24 h and 48 h incubation on pristine polished titanium (pol-Ti), functionalized with an intermediate PDA layer (pDA-Ti) and coated with DNase I by means of AC-EPD (DNase-EPD) or classic dipping (DNase-dip). For each condition and time point, three independent biological replicates were analysed, with three CLSM image areas quantified per replicate, resulting in nine data points per condition. Statistical significance was evaluated using Welch’s t-test and indicated as follows: *p* < 0.05 (*), *p* < 0.01 (**), and *p* < 0.001 (***).

For anchorage-dependent cells, such as keratinocytes, the first biological action upon implantation, i.e., cell adhesion, is crucial and forms a basis for proper cell functioning, including cell proliferation, migration and differentiation (Yao et al., 2013). A stable cell adhesion is a prerequisite for the soft-tissue regeneration after implantation. Moreover, keratinocytes play a significant role in the integration progress of dental implants, establishing a soft-tissue seal that will prevent ingression of microorganisms towards the bone/implant interface. Therefore, in addition to cell viability, HOK cell adhesion was investigated after 24 h and 48 h of incubation using immunofluorescence staining in order to capture phalloidin-positive actin and Hoechst-positive nuclei in order to observe the cell morphology by CLSM (Figure 7). After 24 h of incubation (Figure 7a), less dominant cell spreading was observed on pol-Ti, which slightly improved for pDA-Ti. This is indicative of a limited cell adhesion, which was also reflected in the limited number of adhered cells on both surfaces. In comparison, cell spreading appears distinctly better on DNase-EPD and DNase-dip as seen by the actin surface coverage. Concurrently, more cells adhered to these latter surfaces. Upon a longer incubation time of 48 h (Figure 7b), cell spreading seemed even further reduced on pol-Ti, whereas it was improved on pDA-Ti. PDA layer has been used to enhance cell growth and spreading of various cell types including human umbilical vein endothelial cells, human corneal limbal epithelial cells, or mouse osteoblast MC3T3-E1 (Jeong et al., 2011; Ku et al., 2010; Ku and Park, 2010). Our findings agree with previously reported studies (Merino-Gómez et al., 2023; Steeves et al., 2016) on the incorporation of PDA layer for the enhancement of cell growth and spreading. DNase-EPD and DNase-dip seemed to exhibit a lower cell adherence compared to 24 h incubation. However, statistical analysis confirmed that both PDA-modified and DNase I coated samples supported significantly greater spreading than pol-Ti at 24 h and 48 h. Overall, the actin surface coverage as well as the number of nuclei appears higher for DNase-EPD than for DNase-dip.

To quantify these qualitative observations, CLSM images were used to count the amount of adhered HOKs (via nuclei), as a measure of the cell attachment, and the surface area of actin cytoskeletons, as a measure of the cell spreading (Figures 7c,d). After 24 h incubation, the highest number of adhered cells was observed on DNase-EPD. This increase in attachment was statistically significant when compared to pol-Ti (p = 0.0019), PDA-Ti (p = 0.001), and DNase-dip (p = 0.0028), demonstrating the strong adhesion-promoting effect of this coating. Longer incubation did not result in a further increase of the cell number (Figure 7c). Only pDA-Ti sample enhanced the adhered cell number dependent on incubation time. The rest of the samples, i.e., pol-Ti and DNase-dip, did not show a remarkable change, however, the deviation was much lower for DNase-dip sample after 48 h incubation (Figure 7d). A similar trend was observed for the cell’s surface coverage as calculated from the actin surface area (Figure 7d). At 24 h, DNase-EPD showed significantly higher spreading than pol-Ti (p = 0.0012), PDA-Ti (p = 0.0029), and DNase-dip (p = 0.0209). After 48 h, DNase-EPD maintained superior spreading relative to pol-Ti (p = 0.0024) and DNase-dip (p = 0.035), while the comparison with PDA-Ti showed a non-significant trend (p = 0.13). These results identify DNase-EPD as the statistically best-performing surface in promoting both attachment and spreading at both time points. It has been suggested in the literature that peptide coatings had a stimulatory effect on the proliferation of keratinocytes compared to control surfaces such as etched Ti surface or silanized-Ti surface (Koidou et al., 2018). In this study, cell attachment and spreading were increased on both PDA-functionalized Ti and enzyme coated samples (i.e., DNase-EPD and DNase-dip). PDA layer is known as an effect layer to contribute soft-tissue integration on various substrates (Zhu et al., 2019; Yang et al., 2020; Liu et al., 2015). Incorporation of PDA layer on bio-inert surfaces resulted in an increase number of soft-tissue cells (i.e., human gingival fibroblast), and area of coverage (Yang et al., 2020; Liu et al., 2015), which is also in good agreement with our findings.

**FIGURE 8:**
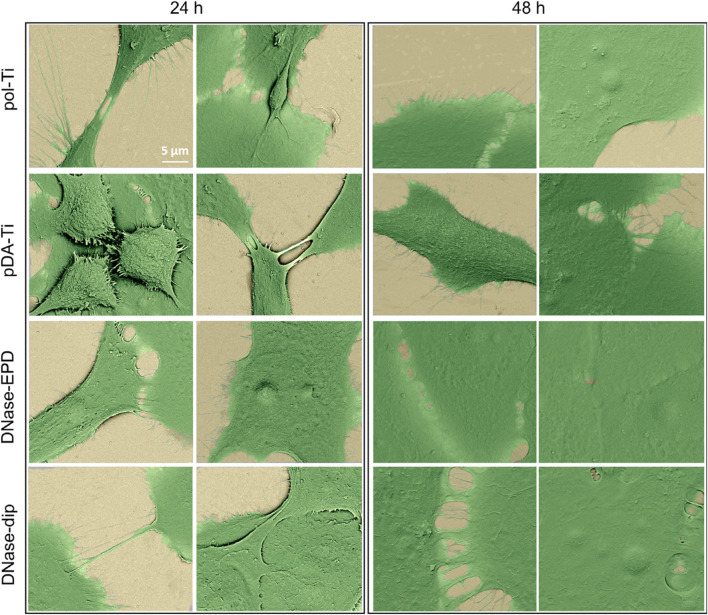
Representative SEM images of human oral keratinocytes (HOKs) on DNase-coated samples after 24 h and 48 h of incubation. The morphology of HOKs after 24 h and 48 h of incubation is shown on pristine polished titanium (pol-Ti), titanium functionalized with an intermediate polydopamine layer (pDA-Ti), and titanium coated with DNase I by AC-EPD (DNase-EPD) or by classic dipping (DNase-dip).

To further investigate the cell morphology in more detail, SEM was performed to image the HOK adhering on different surfaces (Figure 8). HOKs exhibited a spherical shape on pol-Ti and pDA-Ti sample surfaces after 24 h incubation, however, cells membrane protrusion and pseudopodia were more enhanced on DNase-treated samples. Moreover, longer incubation time led to development of more protrusions and pseudopodia of HOK cell membrane on each sample, resulting in an increased coverage. These results are in agreement with a previously reported study on the immobilization of collagen on Ti using a PDA functionalization step as done here as well (Zhu et al., 2019). After 24 h incubation on pristine and PDA-functionalized Ti surfaces, keratinocytes had a spherical keratinocyte appearance, however, on the collagen biomolecule coating formed more protrusions and pseudopodia (Zhu et al., 2019).

Even though previous studies have shown the antimicrobial properties of DNase I coatings against the most common orthopedic pathogens (Aktan et al., 2022a; Swartjes et al., 2013; Ye et al., 2017), recombinant human DNase is also considered as a therapeutic agent to treat life-threatening diseases such as asthma (Silverman et al., 2012; Patel et al., 2000). Overall, the result of the current study opens possibilities for an additional function of DNase I coatings, such as for soft-tissue applications owing to their superior behavior for keratinocytes adhesion. During their lifetime, dental implants are more prone to bacterial attachment than orthopedic implants due to the pathogen-rich oral cavity. As such, there is a need to develop a protective soft-tissue seal at the abutment level to secure the implant from bacterial colonization. If the soft-tissue attachment cannot be completed or stably maintained, bacterial invasion can spread to the osseo-integrated part, which will eventually result in peri-implantitis (Lee et al., 2017). Therefore, dental implant surfaces capable of preventing bacterial colonization and biofilm formation while at the same time supporting soft-tissue integration hold great promise for the prevention of peri-implant diseases (Van Den Borre et al., 2022). The current study into the cytocompatibility of DNase I AC-EPD coating provides a guideline for dental soft-tissue applications.

## 4 Conclusion

In this study, DNase I coatings either prepared by AC-EPD or by dip coating method (i.e., classic dipping) were evaluated in terms of mechanical stability and *in vitro* cytocompatibility. First, mechanical stability was addressed by mechanically challenging the coatings by means of ultrasonication. Water CA measurements showed no significant changes in wettability upon ultrasonication, while ToF-SIMS confirmed that DNase-related fragments on both DNase-treated samples remained present at the surface. However, fresh DNase I coatings exhibited more amino acid fragments compared to samples sonicated for 2 h. The disulfide bond content, indicative of the protein tertiary structure, revealed slight denaturation for DNase-dip coatings as a result of the sonication, whereas for DNase-EPD samples, the disulfide bond content strongly increased. Cytocompatibility tests were performed using HOKs and it could be observed that DNase-coatings did not cause any cytotoxic effects regardless of the coating procedure. The amount of adhered HOKs, as a measure of the cell attachment, and the surface area of actin cytoskeletons, as a measure of the cell spreading, were counted using CLSM images. The highest number of adhered cells was observed on DNase-EPD after 24 h incubation, however, a further increase in the number of adhered cells was not observed for longer incubation. These data were further corroborated by the SEM images where it could be observed that the HOK had developed cell protrusions and pseudopodia when in contact with DNase coatings. Following by 48 h incubation, both DNase-treated surfaces exhibited a complete coverage which was observed by SEM. Overall, these results suggest that PDA-functionalized AC-EPD DNase I coatings are sufficiently stable for a clinical application and besides having antimicrobial properties, support the proper functioning of soft-tissue cells. As such, this new coating system is a promising surface modification strategy for dental implant materials.

## Data Availability

The original contributions presented in the study are included in the article/Supplementary Material, further inquiries can be directed to the corresponding authors.
