# Supplementary material for: Enhancing implant surfaces: mechanical stability and cytocompatibility of DNase I coatings deposited by alternating current electrophoretic deposition
Source: Front Bioeng Biotechnol. 2026 Jan 12;13:1738602. doi: 10.3389/fbioe.2025.1738602 (PMC12832641; doi:10.3389/fbioe.2025.1738602)
Supplement: Supplementary file 1 [file Table1.docx]

Supporting Info

**Enhancing Implant Surfaces: Mechanical Stability and Cytocompatibility of AC-EPD DNase I Coatings**

Merve Kübra Aktan^1,*^, Naiera Zayed^2,3^, Aydan Yadigarli^4^, Manuela Sonja Killian^4^, Rob Lavigne^5^, Wim Teughels^2^, Annabel Braem^1,*^

^1^KU Leuven, Department of Materials Engineering (MTM), Biomaterials and Tissue Engineering Research Group, Leuven, Belgium

^2^ KU Leuven, Faculty of Medicine, Department of Oral Health Sciences, Leuven, Belgium

^3^ Menoufia University, Faculty of Pharmacy, Department of Microbiology and Immunology, Shebin El-Kom, Egypt

^4^ University of Siegen, Chemistry and Structure of Novel Materials, Siegen, Germany

^5^ KU Leuven, Department of Biosystems, Laboratory of Gene Technology, Leuven, Belgium

**Table S1**. Peak list used for PCA analysis of positive polarity spectra [1].

| **Mass** | **Ion** | **Assignment** |
| --- | --- | --- |
| 17.03 | NH_3_^+^ | amino acids |
| 18.03 | NH_4_^+^ | amino acids |
| 22.99 | Na^+^ | salt* |
| 28.02 | CH_2_N^+^ | Leu + others |
| 30.04 | CH_4_N^+^ | Leu, Gly + others |
| 31.02 | CH_3_O^+^ | carboxylic acids |
| 31.04 | CH_5_N^+^ | amino acids |
| 37.01 | C_3_H^+^ | carbohydrates (aromatic) |
| 38.02 | C_3_H_2_^+^ | PDA |
| 38.96 | K^+^ | salt* |
| 39.96 | Ca^+^ | salt* |
| 41.03 | C_2_H_3_N^+^ | Ala, Gly , His, Leu, Ser |
| 42.03 | C_2_H_4_N^+^ | Ala, Gly , His, Leu, Ser |
| 43.02 | C_2_H_3_O^+^ | PDA |
| 43.03 | CH_3_N_2_^+^ | Arg |
| 43.04 | C_2_H_5_N^+^ | Ala, Leu, Ser |
| 43.05 | C_3_H_7_^+^ | carbohydrates |
| 44.01 | CH_2_NO^+^ | PDA |
| 44.04 | CH_4_N_2_^+^ | Arg |
| 44.05 | C_2_H_6_N^+^ | Ala, Asn, Leu |
| 44.98 | CHS^+^ | Cys |
| 44.99 | CHO_2_^+^ | carboxylic acids |
| 45.99 | CH_2_S^+^ | Cys |
| 47.95 | Ti^+^ | substrate* |
| 54.03 | C_3_H_4_N^+^ | His |
| 55.02 | C_3_H_3_O^+^ | Tyr |
| 56.05 | C_3_H_6_N^+^ | Lys, Met, Val |
| 57.05 | C_3_H_7_N^+^ | PDA |
| 57.07 | C_4_H_9_^+^ | carbohydrate |
| 58.06 | C_3_H_8_N^+^ | PDA/Glu |
| 58.99 | C_2_H_3_S^+^ | Cys |
| 59.05 | CH_5_N_3_^+^ | Arg |
| 60.04 | C_2_H_6_NO^+^ | Ser |
| 61.01 | C_2_H_5_S^+^ | Met |
| 63.02 | C_5_H_3_^+^ | carbohydrates (aromatic) |
| 63.94 | TiO^+^ | substrate* |
| 64.95 | TiOH^+^ | substrate* |
| 65.96 | TiH_2_O^+^ | substrate* |
| 68.05 | C_4_H_6_N^+^ | Pro (, Lys) |
| 69.03 | C_4_H_5_O^+^ | Thr |
| 70.03 | C_3_H_4_NO^+^ | Asn |
| 70.07 | C_4_H_8_N^+^ | Pro, Val, Arg, Leu |
| 71.01 | C_3_H_3_O_2_^+^ | Ser |
| 72.04 | C_3_H_6_NO^+^ | Gly |
| 72.08 | C_4_H_10_N^+^ | Val |
| 73.06 | C_2_H_7_N_3_^+^ | Arg |
| 74.06 | C_3_H_8_NO^+^ | Thr |
| 76.02 | C_2_H_6_SN^+^/ C_2_H_6_O_2_N^+^ | Cys/PDA |
| 80.94 | TiO_2_H^+^ | substrate* |
| 82.05 | C_4_H_6_N_2_^+^ | His |
| 83.05 | C_5_H_7_O^+^ | Val |
| 84.04 | C_4_H_6_NO^+^ | Gln, Glu |
| 84.08 | C_5_H_10_N^+^ | Lys, Leu |
| 86.10 | C_5_H_12_N^+^ | Ile, Leu |
| 87.06 | C_3_H_7_N_2_O^+^ | Asn |
| 88.04 | C_3_H_6_NO_2_^+^ | Asn, Asp |
| 91.05 | C_7_H_7_^+^ | carbohydrates (aromatic) |
| 98.02 | C_4_H_4_NO_2_^+^ | Asn |
| 100.09 | C_4_H_10_N_3_^+^ | Arg |
| 101.10 | C_4_H_11_N_3_^+^ | Arg |
| 102.06 | C_4_H_8_NO_2_^+^ | Glu |
| 102.13 | C_6_H_16_N^+^ | PDA |
| 107.05 | C_7_H_7_O^+^ | Tyr |
| 110.08 | C_5_H_8_N_3_^+^ | His, Arg |
| 112.09 | C_5_H_10_N_3_^+^ | Arg |
| 113.11 | C_6_H_11_NO^+^ | PDA |
| 114.13 | C_6_H_12_NO^+^ | PDA |
| 120.08 | C_8_H_10_N^+^ | Phe |
| 127.10 | C_5_H_11_N_4_^+^ | Arg |
| 129.12 | C_5_H_13_N_4_^+^ | Arg |
| 130.06 | C_9_H_8_N^+^ | Trp |
| 130.16 | C_7_H_16_NO^+^ | PDA |
| 131.05 | C_9_H_7_O^+^ | Phe |
| 186.22 | C_8_H_16_N_3_O^+^ | PDA |
| 402.14 | C_22_H_16_N_3_O_5_^+^ | PDA |
| 403.13 | C_22_H_17_N_3_O_5_^+^ | PDA |

**S1. References**

[1] M.S. Killian, A.J. Taylor, D.G. Castner, Stabilization of dry protein coatings with compatible solutes, Biointerphases. 13 (2018) 06E401-8. https://doi.org/10.1116/1.5031189.
